# Family History and Gastric Cancer Risk: A Pooled Investigation in the Stomach Cancer Pooling (STOP) Project Consortium

**DOI:** 10.3390/cancers13153844

**Published:** 2021-07-30

**Authors:** Facundo Vitelli-Storelli, María Rubín-García, Claudio Pelucchi, Yolanda Benavente, Rossella Bonzi, Matteo Rota, Domenico Palli, Monica Ferraroni, Nuno Lunet, Samantha Morais, Weimin Ye, Amelie Plymoth, Reza Malekzadeh, Shoichiro Tsugane, Akihisa Hidaka, Nuria Aragonés, Gemma Castaño-Vinyals, David Georgievich Zaridze, Dmitry Maximovich, Jesus Vioque, Manuela García-de-la-Hera, Zuo-Feng Zhang, Gerson Shigueaki Hamada, Mohammadreza Pakseresht, Farhad Pourfarzi, Lina Mu, Stefania Boccia, Roberta Pastorino, Guo-Pei Yu, Areti Lagiou, Pagona Lagiou, Eva Negri, Carlo La Vecchia, Vicente Martín

**Affiliations:** 1 Grupo de Investigación en Interacciones Gen-Ambiente y Salud (GIIGAS), Institute of Biomedicine (IBIOMED), University of León, 24071 León, Spain; fvits@unileon.es (F.V.-S.); mrubig@unileon.es (M.R.-G.); vmars@unileon.es (V.M.); 2 Department of Clinical Sciences and Community Health, University of Milan, 20133 Milan, Italy; claudio.pelucchi@unimi.it (C.P.); rossella.bonzi@unimit.it (R.B.); monica.ferraroni@unimi.it (M.F.); eva.negri@unimi.it (E.N.); carlo.lavecchia@unimi.it (C.L.V.); 3 Cancer Epidemiology Research Programme, Catalan Institute of Oncology, IDIBELL, Hospitalet de Llobregat, 08908 Barcelona, Spain; 4 Consortium for Biomedical Research in Epidemiology and Public Health (CIBERESP), 28029 Madrid, Spain; nuria.aragones@salud.madrid.org (N.A.); gemma.castano@isglobal.org (G.C.-V.); vioque@umh.es (J.V.); manoli@umh.es (M.G.-d.-l.-H.); 5 Department of Molecular and Translational Medicine, University of Brescia, 25121 Brescia, Italy; matteo.rota@unibs.it; 6 Cancer Risk Factors and Life-Style Epidemiology Unit, Institute for Cancer Research, Prevention and Clinical Network, ISPRO, 08518 Florence, Italy; d.palli@ispro.toscana.it; 7 EPIUnit—Instituto de Saúde Pública da Universidade do Porto, 4050-091 Porto, Portugal; nlunet@med.up.pt (N.L.); samantha.morais@ispup.up.pt (S.M.); 8 Departamento de Ciências da Saúde Pública e Forenses e Educação Médica, Faculdade de Medicina da Universidade do Porto, 4200-319 Porto, Portugal; 9 Department of Medical Epidemiology and Biostatistics, Karolinska Institutet, 17177 Stockholm, Sweden; weimin.ye@ki.se (W.Y.); amelie.plymoth@ki.se (A.P.); 10 Digestive Oncology Research Center, Digestive Disease Research Institute, Tehran University of Medical Sciences, Tehran 14117-13135, Iran; malek@tums.ac.ir (R.M.); mpakseresht@ualberta.ca (M.P.); f.pourfarzi@arums.ac.ir (F.P.); 11 Epidemiology and Prevention Group, Center for Public Health Sciences, National Cancer Center, Tokyo 104-0045, Japan; stsugane@ncc.go.jp (S.T.); ahidaka@ncc.go.jp (A.H.); 12 Cancer Epidemiology Section, Public Health Division, Department of Health of Madrid, 28035 Madrid, Spain; 13 IMIM (Hospital del Mar Medical Research Institute), 08003 Barcelona, Spain; 14 Department of Public health, Universitat Pompeu Fabra (UPF), 08002 Barcelona, Spain; 15 Barcelona Institute for Global Health-ISGlobal, 08036 Barcelona, Spain; 16 Department of Epidemiology and Prevention, Russian N.N. Blokhin Cancer Research Center, 115478 Moscow, Russia; dgzaridze@crc.umos.ru (D.G.Z.); dmax@crc.umos.ru (D.M.); 17 Instituto de Investigación Sanitaria y Biomédica de Alicante, ISABIAL-UMH, 46020 Alicante, Spain; 18 Department of Epidemiology, UCLA Fielding School of Public Health and Jonsson Comprehensive Cancer Center, Los Angeles, CA 90095-6900, USA; zfzhang@ucla.edu; 19 Nikkei Disease Prevention Center, São Paulo 13010-111, Brazil; gehamada@gmx.com; 20 Department of Agricultural, Food and Nutritional Sciences, University of Alberta, Edmonton, AB T6G 2R3, Canada; 21 Nutritional Epidemiology Group, Centre for Epidemiology and Biostatistics, University of Leeds, Leeds LS2 9JT, UK; 22 Digestive Disease Research Center, Ardabil University of Medical Sciences, Ardabil 56189-85991, Iran; 23 Department of Epidemiology and Environmental Health, School of Public Health and Health Professions, University at Buffalo, Buffalo, NY 14261, USA; linamu@buffalo.edu; 24 Section of Hygiene, University Department of Life Sciences and Public Health, Università Cattolica del Sacro Cuore, 20123 Rome, Italy; Stefania.Boccia@unicatt.it (S.B.); Roberta.Pastorino@unicatt.it (R.P.); 25 Department of Woman and Child Health and Public Health—Public Health Area, Fondazione Policlinico Universitario A. Gemelli IRCCS, 00168 Roma, Italy; 26 Medical Informatics Center, Peking University, Beijing 100191, China; yugp@msn.com; 27 Department of Public and Community Health, School of Public Health, University of West Attica, 12243 Athens, Greece; alagiou@uniwa.gr; 28 Department of Hygiene, Epidemiology and Medical Statistics, School of Medicine, National and Kapodistrian University of Athens, 15784 Athens, Greece; plagiou@hsph.harvard.edu; 29 Department of Epidemiology, Harvard T.H. Chan School of Public Health, Boston, MA 01451, USA

**Keywords:** gastric cancer, family history, international consortium, meta-analyses

## Abstract

**Simple Summary:**

Research is still required to establish the relationship between family history (FH) and gastric cancer (GC) in relation to different histological types and anatomical sites. The present work aimed to examine the influence of first-degree FH on the risk of GC, also according to the GC location and histological type, including 5946 cases and 12,776 controls from 17 studies of 11 countries in three continents participating in the Stomach Cancer Pooling (StoP) Project consortium. This analysis confirms the effect of FH on the risk of GC, reporting an approximately doubled risk, and provides further quantification of the risk of GC according to the subsite and histotype.

**Abstract:**

Although there is a clear relationship between family history (FH) and the risk of gastric cancer (GC), quantification is still needed in relation to different histological types and anatomical sites, and in strata of covariates. The objective was to analyze the risk of GC according to first-degree FH in a uniquely large epidemiological consortium of GC. This investigation includes 5946 cases and 12,776 controls from 17 studies of the Stomach Cancer Pooling (StoP) Project consortium. Summary odds ratios (OR) and the corresponding 95% confidence intervals (CIs) were calculated by pooling study-specific ORs using fixed-effect model meta-analysis techniques. Stratified analyses were carried out by sex, age, tumor location and histological type, smoking habit, socioeconomic status, alcohol intake and fruit consumption. The pooled OR for GC was 1.84 (95% CI: 1.64–2.04; I2 = 6.1%, P heterogeneity = 0.383) in subjects with vs. those without first-degree relatives with GC. No significant differences were observed among subgroups of sex, age, geographic area or study period. Associations tended to be stronger for non-cardia (OR = 1.82; 95% CI: 1.59–2.05 for subjects with FH) than for cardia GC (OR = 1.38; 95% CI: 0.98–1.77), and for the intestinal (OR = 1.92; 95% CI: 1.62–2.23) than for the diffuse histotype (OR = 1.62; 95% CI: 1.28–1.96). This analysis confirms the effect of FH on the risk of GC, reporting an approximately doubled risk, and provides further quantification of the risk of GC according to the subsite and histotype. Considering these findings, accounting for the presence of FH to carry out correct prevention and diagnosis measures is of the utmost importance.

## 1. Introduction

Gastric cancer (GC) is the fifth leading cause of cancer by incidence and the third leading cause of cancer death in both sexes worldwide. In 2018, there were an estimated one million new GC cases and nearly 800 thousand deaths [1].

The most accepted model of human gastric carcinogenesis is a multistage model in which both environmental and genetic factors are involved [2]. This includes family history (FH), genetic susceptibility, shared environmental or lifestyle factors and/or a combination of interactions. Between 80 and 90% of GCs are sporadic, 10 and 20% have a positive FH and only between 1 and 3% show a clear Mendelian inheritance pattern [3]. Various studies have investigated the role of FH in relation to GC, often reporting relative risks around or over two for subjects with a positive FH of GC [4]. Such a strong association may be explained, besides the genetic component, by environmental exposures—including smoking habits, diet and particularly *Helicobacter pylori* infection—shared by family members. Still, an unexplained large variability between risk estimates has been reported according to geographic area, ethnic group and sex, as well as the histological type and location of GC [5]. Given the relatively small proportion of GC cases with a positive FH, only a few large studies to date have been able to examine the role of FH on different locations and histological types of GC, as well as in strata of covariates. 

Five-year GC survival in most countries is less than 30% [6]. Therefore, it is important to consider FH in prevention or early detection. The present work aims to examine the influence of first-degree FH on the risk of GC, also according to the GC location and histological type, in 17 studies from 11 countries in three continents participating in the Stomach Cancer Pooling (StoP) Project consortium.

## 2. Materials and Methods

The StoP Project is a consortium of epidemiological studies on gastric cancer. A detailed description of its aims and methods has been provided elsewhere [7]. Inclusion criteria for study participation were: case–control study design, including nested case–control within cohort studies, and inclusion of at least 80 cases of incident, histologically confirmed GC (including both cardia and non-cardia locations). In addition, the original questionnaires and useful information were collected to help with data handling from studies, in order to optimize data harmonization.

For this analysis, the studies were selected from the StoP consortium studies that had both the family history information and the covariates that we used in the models. This work is based on the second data release of the StoP Project, where 17 studies [8,9,10,11,12,13,14,15,16,17,18,19,20,21,22,23,24,25] conducted in 11 countries had data on the FH of GC and were examined, including a total of 5946 cases and 12,776 controls. The following data were extracted: (i) main study variables, including study design, geographic area, study period, single center/multicentric study and study center if multicentric; (ii) relevant covariates, including sex, age, education/social class, body mass index, total alcohol consumption, smoking habit, *H. pylori* infection and consumption of fruit and vegetables; (iii) specific cancer-related variables (for cases only), including cancer subsite and histotype; (iv) FH of GC among first-degree relatives (parents, siblings and offspring). All of the above variables were harmonized centrally according to a pre-specified format. Furthermore, any additional information related to the FH of GC available in each study (e.g., age at GC occurrence in relatives, type of relative affected) was considered.

The StoP Project received ethical approval from the University of Milan Review Board (reference 19/15 on 1 April 2015).

To estimate the association between FH and GC, a two-stage modeling approach was used [26]. First, the association in each study was assessed by calculating the odds ratio (OR) and the corresponding 95% confidence interval (CI) using multivariate logistic regression models including terms for sex, age, socioeconomic level, smoking status and alcohol consumption. In the second stage, summary effect estimates were computed by pooling study-specific ORs using fixed-effect model meta-analysis techniques. Heterogeneity between studies was evaluated using the Q test statistics and quantified using I^2^—that is, the proportion of total variation contributed by between-study variance [27].

To investigate whether the role of FH was heterogeneous across strata of selected covariates, analyses stratified by sex, age, socioeconomic status, GC location (cardias/non cardias), histological subtype (intestinal, diffuse), tobacco smoking, socioeconomic status, alcohol intake, fruit consumption and *H. pylori* infection were carried out. Additional analyses were carried out according to type of study, control selection system (matched or by frequency), source of controls (population or hospital) and study period (XX or XXI century).

## 3. Results

The main characteristics of the StoP Project studies included in the present analysis are shown in Table 1. Most studies were conducted in European countries (82.3% of the controls, and 77.9% of the cases). Overall, 15.8% of cases (*n* = 942) and 7.7% of the controls (*n* = 979) had an FH of stomach cancer in first-degree relatives, ranging from 4.4 to 23.0% among cases, and from 1.0 to 16.8% among controls.

Table 2 shows the frequency distribution of cases and controls according to the selected covariates. Cases were more frequently male (63.7% vs. 58.3%), aged 65 or older (50.5% vs. 43.0%) and of a low socioeconomic status (63.9% vs. 52.1%) compared to controls. Furthermore, cases were more frequently current smokers (28.9% vs. 25.8%) and reported a high alcohol intake (14.5% vs. 10.6%) and a low consumption of fruit (36.3% vs. 29.0%) compared to controls.

**Figure 1:**
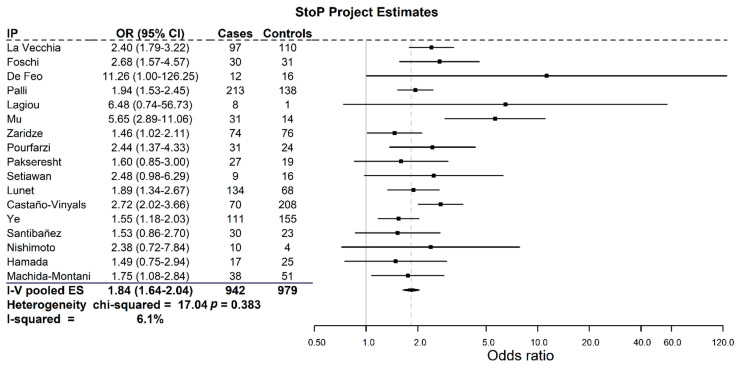
Pooled adjusted odds ratios with 95% confidence intervals for GC related to family history in the StoP Project.

In all studies, first-degree FH was positively related to GC, with ORs ranging between 1.46 and 11.26 (Figure 1). The differences observed were statistically significant in 10 out of the 17 studies included. The pooled OR of all studies was 1.84 (95% CI = 1.64–2.04; I^2^ = 6.1%, P_heterogeneity_ = 0.383). 

**Figure 2:**
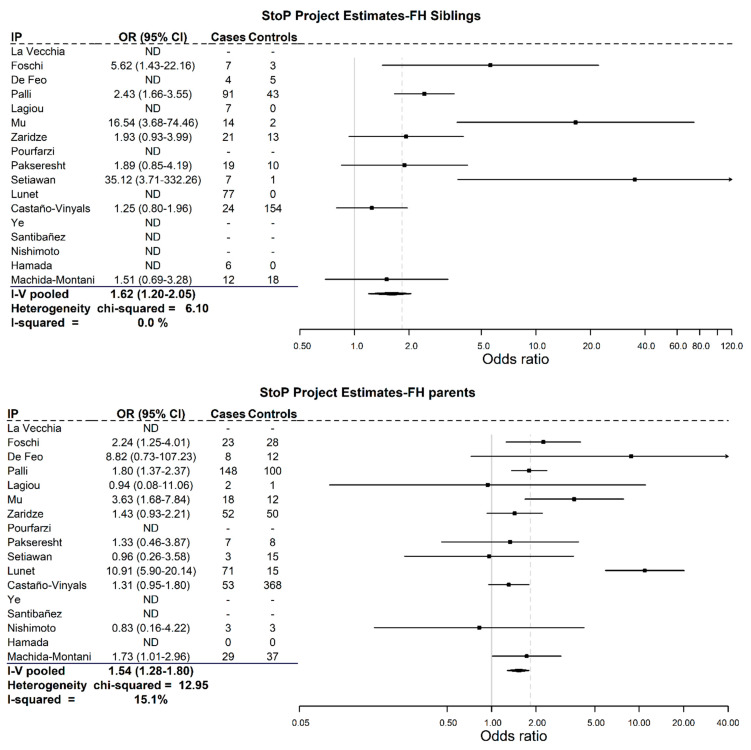
Pooled adjusted odds ratios with 95% confidence intervals considering the type of relative affected.

Regarding the analysis of the type of family member affected, Figure 2 shows that the pooled OR for siblings was higher than for parents (1.62; 95% CI = 1.20–2.05, and 1.54; 95% CI = 1.28–1.80, respectively). No significant heterogeneity was observed.

**Figure 3:**
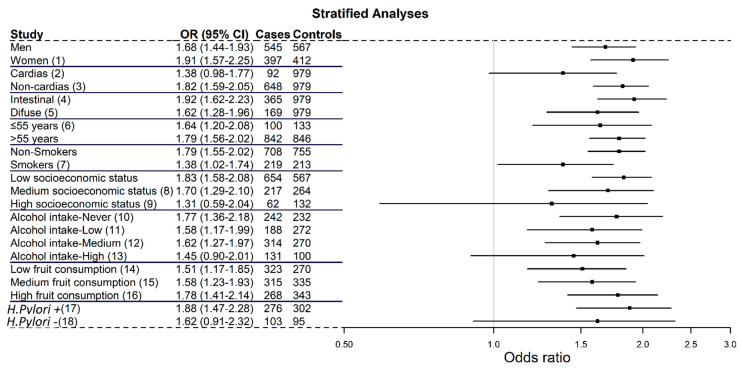
Pooled adjusted odds ratios with 95% confidence intervals for gastric cancer associated with a family history of gastric cancer stratified by sex, age, socioeconomic status, gastric cancer location, histological subtype, tobacco smoking, alcohol intake, fruit consumption and Helicobacter pylori infection in the StoP Project. No information for studies: (1) De Feo, Lagiou; (2) Foschi, Lagiou, Mu, Setiawan; (3) Mu, Setiawan; (4) La Vecchia, Lagiou, Mu, Setiawan, Machida-Montani; (5) La Vecchia, Lagiou, Mu, Setiawan, Machida-Montani; (6) De Feo, Lagiou, Pourfarzi, Pakseresht, Castaño-Vinyals; (7) De Feo, Lagiou, Pourfarzi, Pakseresht, Castaño-Vinyals, Nishimoto; (8) De Feo, Lagiou, Pourfarzi, Pakseresht, Castaño-Vinyals, Nishimoto; (9) De Feo, Palli, Lagiou, Pourfarzi, Setiawan, Santibañez; (10) De Feo, Lagiou, Pakseresht, Setiawan; (11) De Feo, Lagiou, Pourfarzi, Pakseresht, Setiawan, Nishimoto, Hamada; (12) Mu, Pourfarzi, Pakseresht, Setiawan, Nishimoto, Hamada; (13) De Feo, Lagiou, Mu, Pourfarzi, Pakseresht, Setiawan, Ye, Nishimoto, Hamada; (14) Lagiou, Hamada; (15) De Feo, Satiawan, Nishimoto; (16) De Feo, Lagiou, Setiawan; (17) La Vecchia, Foschi, De Feo, Palli, Lagiou, Setiawan, Santibañez; (18) La Vecchia, Foschi, De Feo, Palli, Lagiou, Pakseresht, Setiawan.

Figure 3 shows the results from the stratified analyses. No significant differences were found by sex, +68% in men and +91% in women. Thirteen and fourteen studies provided information on the location of cardia and non-cardia GC, respectively. Of the 695 cardia GC cases, 92 (13.2%) had a first-degree FH of GC, while 648 (17.6%) of the 3676 non-cardia GC cases had a first-degree FH of GC. The pooled ORs were 1.38 (95% CI = 0.98–1.77) and 1.82 (95% CI = 1.59–2.05), respectively. Twelve studies reported information on the histological classification of GC. Of the 2030 intestinal GC cases, 365 (18.0%) had a first-degree FH of GC, yielding a pooled OR of 1.92 (95% CI = 1.62–2.23). The diffuse histological GC type was reported in 1170 cases, with 169 (14.4%) having a first-degree FH of GC. The pooled OR for all studies was 1.62 (95% CI = 1.28–1.96). There were no significant differences according to age, smoking habit, socioeconomic status, alcohol intake, fruit consumption or *H. pylori* infection. 

Furthermore, no significant differences were observed according to whether the studies were performed in European (OR = 1.84, 95% CI = 1.62–2.06) or non-European populations (OR = 1.86, 95% CI = 1.33–2.38); were matched (OR = 1.83, 95% CI = 1.30–2.36) or not-matched (OR = 1.85, 95% CI = 1.63–2.07); and were multicentric (OR = 1.83; 95% CI = 1.57–2.09) or not (OR = 1.87, 95% CI = 1.54–2.21). Modest differences were observed according to the type of controls (hospital-based controls: OR = 1.76, 95% CI = 1.43–2.09; population-based controls: OR = 1.90, 95% CI = 1.64–2.15), and to the study period before the year 2000 (OR = 1.75, 95% CI = 1.51–1.98) or after the year 2000 (OR = 2.11, 95% CI = 1.72–2.50).

## 4. Discussion

The results of this uniquely large collaborative study confirm and quantify the influence of FH on the development of GC better than previously available studies. History of GC in a first-degree relative has been found to increase the risk of GC by about 85%. In this pooled investigation, results were suggestive of a possible higher risk of non-cardia than of cardia GC in subjects with a positive FH of gastric cancer, whereas no relevant differences were observed in strata of sex, in the histological type of GC or in the characteristics analyzed in epidemiological studies. 

The family aggregation of GC is due to a complex interaction between genetic inheritance and environmental and lifestyle factors [28]. It is known that between 10 and 20% of people who develop GC have a FH, but only part of this can be attributed to hereditary syndromes. The three primary familial gastric cancers include hereditary diffuse gastric cancer [3], familial intestinal gastric cancer and gastric adenocarcinoma and proximal polyposis of the stomach, caused by germline mutations in genes such as CDH1, CTNNA1 and APC [29]. The remaining part may be due to low-penetrance genes, which, due to their interaction with the family-shared environment, have an important influence [30]. In this way, the genetic inheritance, the unique environment of each individual and the aforementioned family-shared environment can be divided into three pathways of GC development. A study conducted among twins found that these causes account for 28%, 62% and 10% of the variation in GC susceptibility, respectively [31].

Our results do not show relevant differences according to sex. Though findings of different studies have varied, with some of them reporting a stronger risk related to FH among women [32], our finding is broadly consistent with the conclusions of several reviews [8,11,16,20]. Additionally, our results on the GC subsite are consistent with those from a meta-analysis, indicating that FH has a greater relative risk (RR) on non-cardia GC (RR = 1.97; 95% CI = 1.72–2.25) than on cardia GC (RR = 1.46; 95% CI = 0.89–2.39) [28]. Kharazmi et al. also showed similar results in a nationwide Swedish cohort study, where the standardized incidence ratio (SIR) of cardia GC associated with a positive FH of cancer at the same subsite was lower (SIR = 1.70; 95% CI = 1.10–2.50) compared to non-cardia subclasses—antrum (SIR = 5.5; 95% CI = 2.4–11.00) and body (SIR = 4.6; 95% CI = 2.6–7.4) [33]. A large percentage of non-cardia GC cases are attributed to *H. pylori* infection and, therefore, are more likely associated with family transmission [33]. On the other hand, cardia GC is more likely related to lifestyle factors, with issues such as obesity (increases the risk of cardia GC by 82%) [34,35,36], gastroesophageal reflux (increases the risk two to four times) [19,37] and tobacco smoking [38].

The results of the present study show a higher risk of GC when the affected relatives were siblings rather than parents, which is in line with previous studies reporting that the association tends to be stronger among siblings than between parents and offspring [5,28,39,40]. However, one of the limitations of our study is that neither offspring nor the age at diagnosis of the affected family member could be included due to the small sample available for these specific analyses.

Regarding the histological type of GC, our results show that a FH of GC is associated with both intestinal and diffuse subtypes, which is consistent with findings from the prospective Alpha-Tocopherol, Beta-Carotene (ATBC) Cancer Prevention Study [28], which suggested similarly increased risks in both histological subtypes of GC (intestinal: HR 1.53, 95% CI 0.92–2.55; diffuse: HR 1.47, 95% CI 0.72–3.03). However, by taking advantage of the study consortium approach, our findings were based on a larger number of cases for both histotypes, and therefore our estimates are more robust.

*H. pylori* infection is the major environmental factor in gastric carcinogenesis. As in our study, another investigation [41] showed that patients with an FH and *H. pylori* infection had a slightly higher risk of GC than those without *H. pylori* infection. However, these differences were not significant.

No significant differences were found when the geographical origin of the samples of the studies included (European vs. non-European populations) was analyzed. Most previous studies on FH and GC were conducted in Asian countries, in North America and in Northern Europe [9], and this analysis has the advantage of including studies from less frequently considered areas (i.e., eight studies were from the Mediterranean region, two from Iran and two from Brazil). Furthermore, the pooled analysis patient-level approach allows for a direct comparison between estimates of different geographic areas, by limiting methodological variation (through centralized data harmonization, using the same adjustment terms in the models, etc.).

Only moderate differences were observed in relation to studies based on hospital- and population-based controls. A higher risk of GC related to FH emerged for studies conducted after the year 2000, as compared to those conducted earlier. This may be due to a decrease in the weight of environmental factors, particularly *H. pylori* infection and tobacco smoking, with respect to FH [42,43].

As strengths of the study, there are 5946 cases and 12,776 controls available for the analysis, accompanied by a wide variety of predictor variables, providing an adequate statistical power. Furthermore, this project is a collaborative framework, contributing with varied geographical origins. In addition, we assessed the relationship between FH and GC by location and histological types. The most important difference between our work and the rest of the studies and meta-analyses is that, in our case, we worked with a consortium of studies, meaning we directly used the study data instead of the ORs of the published articles. This allows us to adjust all studies for the same variables and to have more homogeneity in the results.

## 5. Conclusions

Our results confirm and further quantify the effect of FH on the development of GC. Subjects with an FH of GC among first-degree relatives have an approximately doubled risk of GC occurrence. It is important to take into account the presence of an FH to carry out GC prevention measures, both primary and secondary, i.e., early diagnosis.

## Figures and Tables

**Table 1:** Main characteristics of the StoP Project studies included in the analyses.

StoP Study ID	Study Area(s)	Period	Study Type	Cases		Controls		References
				With FH	Total	With FH	Total	
1	Milan, Italy	1985–1997	CC, hospital-based	97	768	110	2081	La Vecchia et al. [8]
3	Milan, Italy	1997–2007	CC, hospital-based	30	230	31	547	Foschi et al. [9]
4	Rome, Italy	2006–2010	CC, hospital-based	12	152	16	411	De Feo et al. [10]
5	Four areas, Italy	1985–1987	CC, population-based	213	1016	138	1159	Palli et al. [11]
6	Athens, Greece	1981–1984	CC, hospital-based	8	86	1	97	Lagiou et al. [12]
8	Taixing, Jiangsu, China	2000	CC, population-based	31	206	14	415	Mu et al. [13]
9	Moscow, Russia	1996–1997	CC, hospital-based	74	433	76	593	Zaridze et al. [14]
10	Ardabil, Iran	2004–2005	CC, population-based	31	217	24	394	Pourfarzi et al. [15]
11	Ardabil, Iran	2005–2007	CC, population-based	27	286	19	304	Pakseresht et al. [16]
13	Yangzhong, China	1995	CC, population-based	9	133	16	433	Setiawan et al. [17]
17	North of Portugal	2001–2006	CC, population-based	134	584	68	612	Lunet et al. [25]
21	Ten provinces, Spain	2008–2013	CC, population-based	70	435	208	3418	Castaño-Vinyals et al. [18]
22	Five counties, Sweden	1989–1995	CC, population-based	111	561	155	1164	Ye et al. [19]
23	Two provinces, Spain	1995–1999	CC, hospital-based	30	367	23	433	Santibañez et al. [20]
28	Brazil-Brazilian origin	1991–1994	CC, hospital-based	10	226	4	226	Nishimoto et al. [22]
29	Brazil-Japanese origin	1991–1994	CC, hospital-based	17	93	25	186	Hamada et al. [23]
30	Japan	1998–2002	CC, hospital-based	38	153	51	303	Machida-Montani et al. [24]

**Table 2:** Distribution of cases and controls according to selected covariates.

Variables	Cases		Controls	
	*n*	%	*n*	%
**Sex**				
Male	3790	63.7	7454	58.3
Female	2156	36.3	5322	41.7
**Age**				
18–49	759	12.8	2553	20.0
50–65	2184	36.7	4724	37.0
65–74	2171	36.5	3940	30.8
75–106	832	14.0	1557	12.2
Missing	0	0.0	2	0.0
**Socioeconomic status**				
Low	3799	63.9	6488	50.8
Medium	1543	26.0	3760	29.4
High	501	8.4	2198	17.2
Missing	103	1.7	330	2.6
**Smoking habit (1)**				
Current	1720	28.9	3255	25.5
No	4132	69.5	9386	73.4
Missing	94	1.6	135	1.1
**Alcohol intake g of ethanol/day**				
Never	1526	25.7	3375	26.4
Low (≤12)	1190	20.0	3468	27.1
Intermediate (>12–47)	1745	29.4	3047	23.9
High (>47)	860	14.5	1353	10.6
Missing	625	10.5	1533	12.0
**Fruit intake (2)**				
Low	2157	36.3	3701	29.0
Medium	1872	31.5	4109	32.2
High	1685	28.3	4293	33.6
Missing	232	3.9	673	5.3
***H. pylori* infection**				
Positive	688	11.6	3662	28.7
Negative	1661	27.9	1252	9.8
Missing	3597	60.5	7862	61.5

(1) No: never and former; (2) defined according to study-specific tertiles.

## Data Availability

Not applicable.
